# Modeling metabolic networks of individual bacterial agents in heterogeneous and dynamic soil habitats (IndiMeSH)

**DOI:** 10.1371/journal.pcbi.1007127

**Published:** 2019-06-19

**Authors:** Benedict Borer, Meriç Ataman, Vassily Hatzimanikatis, Dani Or

**Affiliations:** 1 Department of Environmental Systems Science, ETH Zurich, Zurich, Switzerland; 2 Laboratory of Computational Systems Biotechnology, EPFL, Lausanne, Switzerland; University of Wisconsin-Madison, UNITED STATES

## Abstract

Natural soil is characterized as a complex habitat with patchy hydrated islands and spatially variable nutrients that is in a constant state of change due to wetting-drying dynamics. Soil microbial activity is often concentrated in sparsely distributed hotspots that contribute disproportionally to macroscopic biogeochemical nutrient cycling and greenhouse gas emissions. The mechanistic representation of such dynamic hotspots requires new modeling approaches capable of representing the interplay between dynamic local conditions and the versatile microbial metabolic adaptations. We have developed IndiMeSH (Individual-based Metabolic network model for Soil Habitats) as a spatially explicit model for the physical and chemical microenvironments of soil, combined with an individual-based representation of bacterial motility and growth using adaptive metabolic networks. The model uses angular pore networks and a physically based description of the aqueous phase as a backbone for nutrient diffusion and bacterial dispersal combined with dynamic flux balance analysis to calculate growth rates depending on local nutrient conditions. To maximize computational efficiency, reduced scale metabolic networks are used for the simulation scenarios and evaluated strategically to the genome scale model. IndiMeSH was compared to a well-established population-based spatiotemporal metabolic network model (COMETS) and to experimental data of bacterial spatial organization in pore networks mimicking soil aggregates. IndiMeSH was then used to strategically study dynamic response of a bacterial community to abrupt environmental perturbations and the influence of habitat geometry and hydration conditions. Results illustrate that IndiMeSH is capable of representing trophic interactions among bacterial species, predicting the spatial organization and segregation of bacterial populations due to oxygen and carbon gradients, and provides insights into dynamic community responses as a consequence of environmental changes. The modular design of IndiMeSH and its implementation are adaptable, allowing it to represent a wide variety of experimental and *in silico* microbial systems.

## Author summary

Soil bacterial communities are key players in global biogeochemical cycles and drive other soil regulatory and provisional ecosystem functions. Despite the relatively high bacterial abundance found in fertile soil, bacteria occupy only a small fraction of the soil surfaces and often form hotspots with disproportionate contributions to observed biogenic fluxes. As soil opacity and complexity limit detailed observations of such hotspots *in situ*, we have developed a modeling platform, IndiMeSH (Individual-based Metabolic network model for Soil Habitats), to enable systematic study of dense multispecies bacterial communities within a structured habitat resembling (but not limited) to soil. The model is capable of representing multispecies trophic interactions and spatial self-organization in response to nutrient gradients, as confirmed in comparison with published results. IndiMeSH offers new opportunities for quantifying bacterial hotspot formation and dynamics and observe their resilience and response to perturbations in hydration and nutrient conditions.

## Introduction

Soil hosts the greatest diversity and abundance of microbial life in all of the biosphere, with over 10^9^ cells per gram of soil [1] expressing thousands to millions of different operational taxonomic units in small volumes [2,3]. Nonetheless, considering the vast surface area per volume of a typical soil, bacterial cells are sparsely distributed within the soil matrix, covering less than 1% of the total available soil surface [4,5]. This highly non-uniform distribution is further reinforced by localized resource distribution giving rise to microbial hotspots, such as in soil aggregates, the rhizosphere, biopores or the detritusphere [6]. These hotspots are important for large-scale biogeochemical cycles of carbon and nitrogen and for the emission of greenhouse gases [6–9]. Improved prediction of soil ecological functioning mediated by bacterial processes requires better understanding of how these bacterial hotspots function and their response to dynamic conditions and integrated influence on large-scale processes.

In addition to its inherent complexity, soil opacity limits direct visualization of bacterial distribution within soil [10,11]. Although the use of soil thin sections and artificial micrometric pore networks offers insights into spatial quantification of bacterial distributions and self-organization [12,13], the simultaneous acquisition of the dynamic chemical landscape is not yet resolvable. Mechanistic models are capable of bridging some of the experimental gaps and facilitate systematic studies of the interplay of physical, chemical and biological factors and their spatiotemporal interactions. Individual-based models (IBM) have gained in popularity owing to their local scale and cell-level representation of interactions, which offer a useful platform for prediction of emergent spatial patterns and population abundances arising from microscale interactions. IBMs have been used to investigate a range of questions in microbial ecology such as altruism in biofilms [14], the influence of metabolic switching on biofilm structure [15], bacterial coexistence due to hydration dynamics [16], bacterial spatial organization in soil aggregates [17], and the dynamics of bacterial community diversity in desert soils [18]. A common feature of IBM models is the estimation of individual bacterial growth rates and nutrient consumption based on simple growth kinetics (e.g. Monod kinetics) with empirically prescribed stoichiometric parameters. A flexible approach to calculating bacterial cell response to local conditions and potential switching of metabolic strategies became available with the solution of metabolic networks that make no prior assumptions about growth rates [19].

New approaches using spatiotemporal metabolic modeling with genome-scale metabolic networks enable quantification of cell growth rates, and the associated uptake of nutrients and excretion of intermediate (by-product) metabolites. Such approaches have been used to create biofilm models through an extension of Matlab with NetLogo named MatNet [20], a population-based model of trophically interacting bacterial communities [21], an extension of the MatNet approach to include multispecies communities [22], as well as to investigate bacterial intra-colony nutrient heterogeneities [23]. Common to these modeling approaches is their ability to describe trophic dependencies of microbial species that automatically adapt to spatially and temporally varying nutrient conditions. However, all of the above-mentioned spatiotemporal metabolic models employ basic and unresolved representations of the physical microbial habitats that are considerably simpler than conditions in soil. To realistically simulate bacterial life in complex and partially saturated habitats, such as those found in soil, a description of the physical (pore spaces and surfaces) and characteristics of the aqueous habitats with the resulting diffusion fields is essential [5].

In this study, we developed and tested a new type of spatiotemporal metabolic model that assigns metabolic networks to individual agents capable of chemotactic motion within spatially resolved soil habitats. The physical domain is comprised of angular pore networks representing soil pores. The hydration conditions are imposed by a prescribed matric potential that interacts with the physical domain and results in aqueous phase configurations similar to those expected in soil (with dual occupancy of the liquid and gas phases in angular pores). The matric potential is a component of the total soil water potential attributed to capillary and adsorptive forces between water and the soil solid matrix, with values ranging from 0 for saturated soil to large negative as the soil dries (often expressed as negative pressure). The physical domain including the water phase serve as a backbone for numerical diffusion of nutrients and intermediate metabolites, with explicit account of diffusive bottlenecks for oxygen when pores are saturated or for nutrient diffusion in unsaturated soil, whilst maintaining connectivity of the aqueous phase throughout the simulated domain. Finally, bacterial cells are represented as individual agents with the ability to autonomously move and disperse, intercept nutrients and grow at rates determined by local (often spatially variable) nutrient conditions owing to the integration of metabolic networks. The model is compared to predictions obtained from a previously published population-based spatiotemporal metabolic network [21] and to bacterial spatial segregation observations from experimental data and simulation results from a previous IBM [13]. Additionally, the model was used to mimic experiments of glucose perfusion in soil aggregates [24] and strategically to highlight the influence of pore size distributions under wet and dry conditions with contrasting results–a primary distinction between IndiMeSH and other spatiotemporal metabolic network models.

## Methods

**Fig 1:**
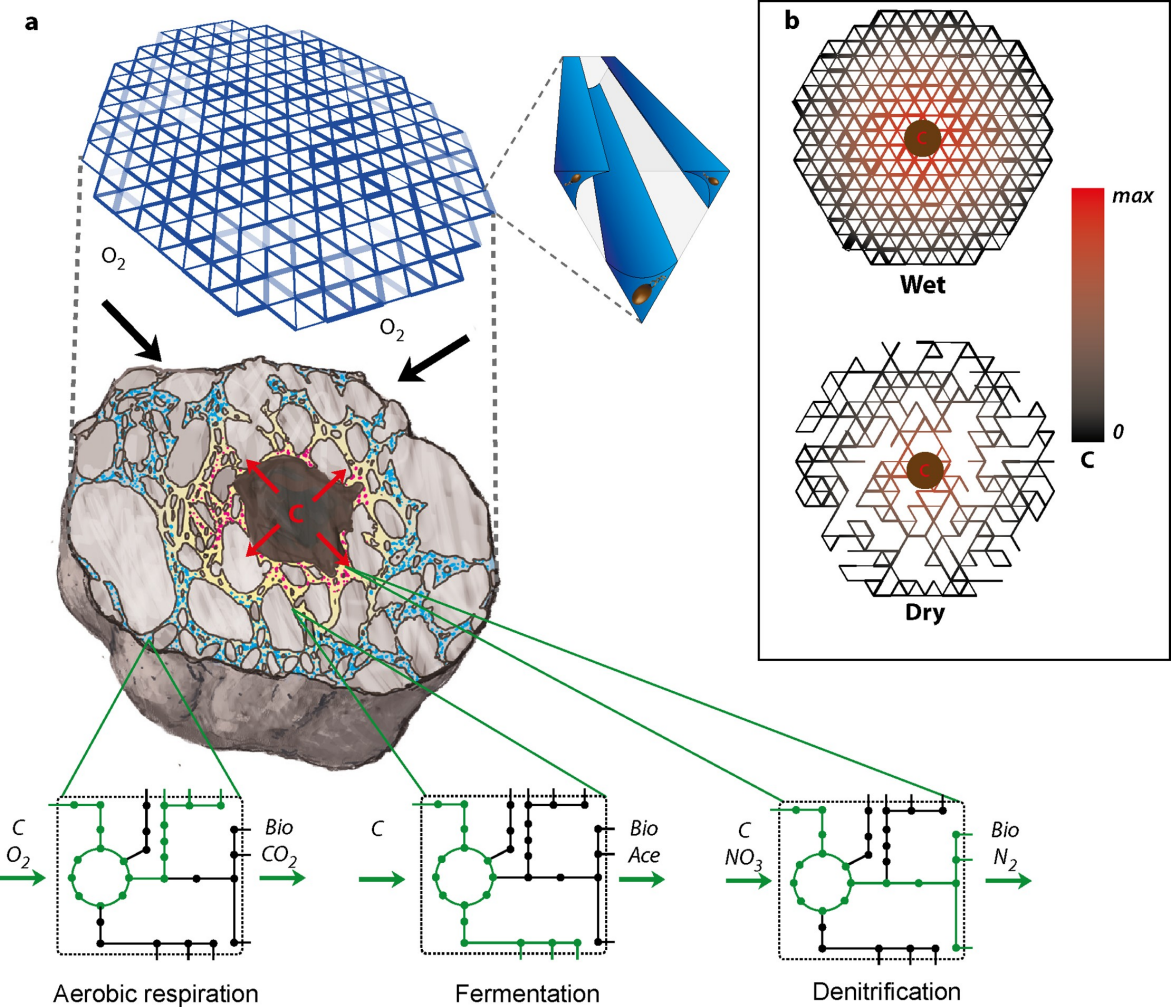
Conceptual image of IndiMeSH model components and methods. **a)** IndiMeSH uses two-dimensional angular pore networks representing soil cross-sections (here a soil aggregate) as a backbone for the aqueous phase configuration, chemical diffusion and as a habitat for bacteria represented as individual agents. Angular pores enable dual occupancy of both the water and gas phase within individual pores. Growth and nutrient consumption is calculated using metabolic networks, which enables the triggering of fundamentally different growth strategies depending on local nutrient and redox conditions. b) For the same pore network, aqueous phase configuration governed by the matric potential (wet and dry conditions) determines the connectivity and thus bacterial dispersal opportunities as well as nutrient diffusion characteristics by limiting water films held within the angular pores.

The purpose of IndiMeSH is to quantify interactions among individual bacterial agents and their immediate environment within a realistic depiction of soil domains and unsaturated conditions. Specifically, we selected bacterial life in pseudo two-dimensional pore networks to mimic processes in cross-sections of soil aggregates and on other soil hydrated rough surfaces. We employ flux balance analysis (FBA) [25] to calculate local nutrient consumption and growth rates along with by-product formation of the motile bacterial agents within the pore network. The heterogeneous nutrient diffusion field and local trophic strategies (consumption and excretion) activate the multifaceted metabolic capabilities of the soil bacterial species under consideration; a conceptual representation of the model is depicted in Fig 1. The model contains four domains: a physical domain containing the actual pore structure and network topology, a (micro)hydrological domain to represent pore water state and properties, a chemical domain simulating nutrient concentrations and diffusion, and a biological domain containing bacterial species traits and properties of individual bacterial agents. These components are described in more detail below. The model is written in the Matlab programming language, computationally optimized to run on laptops and has been developed for users with limited to no programming background.

### The physical domain: Pore network topology and individual pore architecture

A pseudo two-dimensional pore network composed of interlinked pores connected by nodes forming a lattice defines the spatial component of the model. The coordination number expresses the number of pores connected to each node within the lattice (e.g. 4 for a chessboard, 6 for a honeycomb). Each pore has an angular cross-sectional area represented by either isosceles triangles [17] or rectangles (when used to represent microfluidic networks [13]). Angular pores were selected due to their ability to simultaneously have liquid and gas phases under unsaturated conditions, an attribute not possible within cylindrical pores [26]. Different pore sizes are included by varying the inscribed circle radius of each individual pore (for example taken from a lognormal distribution to represent soil pores [17]). The geometry of the triangle was determined by varying the central angle of the isosceles triangle. For rectangular pores, the width and height can be defined for each individual pore (more detail is provided in [17] and [13]). A visual representation of parameters determining pore geometry is provided in S1 Fig.

### The hydrological domain: Water saturation and film thickness

An important aspect of the model is its ability to represent different hydration conditions using the energy state of water or the matric potential. The matric potential determines the aqueous phase configuration (amount retained in the pores and connectivity), hence it also shapes the nutrient diffusional landscape and constrains bacterial dispersal ranges. The water-filled cross-sectional area in each pore is calculated from the matric potential and geometrical considerations [27] as shown in Eq 1. Here A_w_ is the water filled cross-sectional area (m^2^), A_t_ the total cross-section area of the pore (m^2^), Ψ the matric potential (Pa), σ the water surface tension (Nm^-1^) and γ_i_ the angle i within the corner of the angular pore.

$$A_{w}=\left\{ \begin{array}{c} \frac{\sigma^{2}}{\Psi^{2}}*\sum_{i=1}^{n}\left(\frac{1}{\tan\frac{\gamma_{i}}{2}}-\frac{\pi (180-\gamma_{i})}{360}\right),A_{w}<A_{t}\\ \;A_{t},A_{w}\geq A_{t} \end{array}\right.$$ (1)

Pores are considered unsaturated whenever an air phase is present, specifically, when a continuous connection of unsaturated pores is available from a liquid-air interface at the boundary to the pore under consideration. We note that the water-filled cross-sectional area of a pore defines available pathways for nutrient diffusion and for the calculation of total nutrient storage in each pore (depending on pore size, length, and local nutrient concentration). An important parameter of the aqueous phase in each pore is the effective water film thickness defined as the diameter of the largest sphere fully inscribed between the liquid-air interface and the pore wall [28] and calculated using Eq 2. Here, WFT is the water film thickness defined as the smallest radius of an inscribed circle between the liquid-air interface and the pore wall (m), Ψ the matric potential (Pa), σ the water surface tension (Nm^-1^) and γ^i^ the angle within the corner under consideration of the angular pore.

$$WFT=2*\frac{\sigma^{2}}{\Psi^{2}}*\frac{1-\sin\frac{\gamma_{i}}{2}}{1+\sin\frac{\gamma_{i}}{2}}$$ (2)

The water film thickness affects the speed and range of bacterial motility, as further described below. Of particular importance is the fact that different angular pore geometries (e.g., pores with obtuse angles) may retain water films that are too thin to support bacterial flagellated motion, hence, disconnect this bond from other parts of the network in terms of cell dispersion [29]. Despite considerable simplifications compared to natural pore networks, this formulation of physical pore network architecture and associated water configurations retains the salient features of the soil aqueous phase, such as continuity of liquid pathways for nutrient diffusion and cell dispersion and dual occupancy of the liquid and gas phases within angular pores.

### The chemical domain: Nutrient diffusion and boundary conditions

Diffusive fluxes of dissolved substrates within the aqueous phase are described by Fick’s law and are evaluated between nodes subject to mass balance at the nodes, as given by:

$$V_{w}\frac{\partial C}{\partial t}=A_{w}D\frac{\partial^{2}C}{\partial x^{2}}-R$$ (3)

where C is the substrate concentration (M), D the diffusion coefficient in water (m^2^s^-1^), A_w_ the liquid cross-sectional area (m^2^), V_w_ the volume of water attributed to the node (m^3^), x the spatial coordinate along pores (m), and R a sink term (mols^-1^) attributed to the combined consumption of individual bacterial cells. The volume at each node is calculated using Eq 4 where V_w_ the volume of water attributed to the node (m^3^), A_w_ the liquid cross-sectional area of connected pore j (m^2^), L_j_ the length of the connected pore j (m) and d the degree of the node.

$$V_{w}=\sum_{j=1}^{d}\frac{A_{w,j}*L_{j}}{2}$$ (4)

Liquid films of pores sharing the same node are assumed to be continuous. Neglecting bacterial consumption, which is calculated separately using FBA, Eq 1 is solved numerically using an implicit scheme. Nutrient sources are represented by fixed concentration boundaries (e.g. calculated maximum saturation for oxygen using Henry’s law). Consumption and production of nutrients is calculated as the sum of contributions from individual bacterial cells and subtracted from the local nutrient content before calculation of diffusion.

### The biological domain: Bacterial dispersal, local growth and nutrient consumption

Bacteria are represented as individual agents capable of dispersing (by motility when the aqueous film thickness permits) within the pore network, intercepting nutrients and growing according to locally available nutrient conditions. Bacterial cells respond locally and independently with no knowledge of global system properties such as concentration fields or bacterial density even at the nearest pore. Thus, global phenomena such as exponential population growth, spatial organization of bacterial species or diffusion fields due to bacterial consumption are emergent properties based on the local interactions of individual agents with their immediate chemical and physical surroundings. Bacterial cells divide when they reach a prescribed mass at division; they may be removed from the simulation if they deplete internal energy and reach a critical biomass [30].

#### Bacterial growth and nutrient consumption

We employ dynamic Flux Balance Analysis (dFBA) to quantify growth of bacterial species depending on their physiological traits and local nutrient concentrations [21–23,31]. In this study, we have not included intra-species variations, a simplification that enables bacterial biomass to be integrated at each pore. Therefore, we solve the dFBA for each node instead of for each individual bacterial cell, resulting in a significant improvement in computational efficiency. Nutrient uptake rates at each node are calculated based on local concentrations, the volume of water and bacterial biomass. Uptake rates are constrained to the maximum rate specified for each species and specific substrate based on literature values (details stated in S1 Table). Growth rates predicted by FBA are used to calculate the biomass gain for each individual cell using Eq 2, where m_t_ and m_t+1_ are the cell masses (kg) at time step t and t+dt, respectively, μ the growth rate (s^-1^) calculated using FBA, and dt the numerical time step (s). Finally, bacterial nutrient consumption is calculated from the substrate uptake rate per dry weight of cell predicted by dFBA and the local biomass.

$$m_{t+1}=m_{t}+\mu *m_{t}*dt$$ (5)

#### Bacterial motility and chemotactic sensitivity

Bacterial motility is an important aspect of local interactions and self-organization. Chemotactic motility is implemented as a series of a run-and-tumble steps [32] where cells run in a straight direction (along the pore) at a constant velocity, followed by a tumble that changes their current direction at random. Within the mathematical model, runs and tumbles occur within the same time step since tumbling time is far shorter than the average run time (approximately an order of magnitude [32]) and are calculated for each individual cell. Bacterial cells run along one-dimensional pores at a constant velocity determined by the local water film thickness [33,34] calculated using Eq 6 where V is restricted bacterial swimming velocity (ms^-1^), V_0_ unrestricted bacterial swimming velocity, F_m_ the flagellar propulsion force (N), F_λ_ the cell-surface hydrodynamic interactions (N) and Fc the capillary pinning force (N). The derivation of this Equation has been previously published [34] and relies primarily on simple geometrical considerations.

$$V=\left\{ \begin{array}{c} V_{0}\frac{F_{m}-F_{\lambda }-F_{c}}{F_{m}},F_{m}-F_{\lambda }-F_{c}>0\\ \;0,F_{m}-F_{\lambda }-F_{c}\leq 0 \end{array}\right.$$ (6)

Chemotaxis enables to bias the resulting swimming direction towards favorable conditions (e.g. aerotaxis towards oxic conditions). This process is included in the mathematical model by calculating the tumbling probability based on the growth rate gradient of individual cells by comparing individual growth rates in time step t and t-1 [18] and is calculated using Eq 7. Here, p_t_ is the tumbling probability with chemotaxis, p_0_ the tumbling probability in absence of any chemotactic behavior, X the chemotactic sensitivity (m^2^s^-1^), v the swimming velocity depending on hydrodynamic interactions (ms^-1^), u_max_ the maximum growth rate (s^-1^) and Δμ the experienced growth rate gradient (m^-1^s^-1^).

$$p_{t}=p_{0}e^{\frac{-X}{2v\mu_{max}}\Delta \mu }$$ (7)

A binomial distribution based on the individual tumbling probabilities is used to determine whether a cell tumbles in the current time step. For tumbling within a pore, a cell has a 50% probability to change direction. When reaching a node, a new direction is selected at random from all connected pores. When entering a pore where the cell would be immobile due to thin water films, the cell has a 10% chance at each time step to detach back into the previous node. Mounting evidence suggests that motile cells can extract themselves from tight spots [35] but information is insufficient to quantify the efficiency of such processes. We thus have assigned a small probability to account for the occurrence of such relatively rare events.

### Simulation details: Metabolic networks and boundary conditions

We evaluated the proposed IndiMeSH framework by comparing it to a population-based metabolic model (COMETS) [21] and to experimental data [13], and used it to mimic experiments of glucose perfusion in soil aggregates [24] to evaluate the potential of IndiMeSH to predict dynamic shifts in the total abundance of a bacterial community due to boundary condition perturbations. In addition, simulations with contrasting hydration conditions and varying pore size distributions were created to highlight the capability of IndiMeSH to simulate bacterial life in soil. A list of all user defined parameters can be found in S2 Table in the supplementary material.

#### Metabolic network reduction

To reduce the computational burden of the simulations and to focus on related parts of the metabolism, genome-scale metabolic networks (GEM) were systematically reduced using two previously published algorithms, redGEM [36] and lumpGEM [37]. These semi-automatic algorithms make use of graph-based search algorithms and user defined information (subsystem of interest, possible carbon sources and potential excreted metabolites) to identify core reactions and metabolites of interest and then create lumped reactions using mixed-integer linear programming that are balanced concerning all intermediate precursors and cofactors along the synthesis pathways. On average, this reduced the number of reactions within the metabolic networks by an order of magnitude, as shown in Table 1. The consistency of the reduced scale metabolic network (rGEM) with its GEM were evaluated by a scheme based on flux variability analysis (FVA) [38]. A similar approach has been used previously to compare an ad hoc reduced network to a genome-scale network, albeit considering only one physiological state of the population [39]. Since we are interested in a wide range of environmental conditions, we compared FVA predictions with varying percentages (100%, 60% and 20%) of the maximum uptakes rates for each substrate. For example in the case of glucose, uptake rates were constrained to 8 mmol/gDW/h, 4.8 mmol/gDW/h and 1.6 mmol/gDW/h. These values were combined with percentages of all other substrates (e.g. with maximum uptake rates of 30 mmol/gDW/h, 18 mmol/gDW/h and 6 mmol/gDW/h in the case of oxygen), resulting in multiple conditions such as glucose limitation in oxygenated environments or glucose abundance in oxygen-limited environments. For each condition, FVA was calculated for both the genome-scale and reduced-scale model, and the predicted flux envelopes for each of the common reactions was compared to observe the percentage of reactions carrying similar fluxes. This enabled a more precise comparison of genome-scale to reduced-scale metabolic models depending on nutrient conditions, and resulted in a total of 81 and 27 conditions for the case of four and three substrates, respectively. Acetate, methionine and oxygen were used for comparing the reduced- and genome-scale metabolic models of Escherichia coli and Salmonella enterica (with additional lactose in the case of E. coli). For Pseudomonas putida and P. stutzeri, the carbon sources citrate and acetate as well as oxygen were included (with additional nitrate in the case of P. stutzeri). These nutrients were chosen due to their availability in the simulation scenarios.

**Table 1:** Summary of genome-scale and reduced-scale metabolic network dimensions for all species. For all species, the number of reactions and metabolites were reduced by an order of magnitude using the redGEM algorithm [36]. This reduced the computational burden significantly.

	Genome-scale model		Reduced-scale model	
	*Metabolites*	*Reactions*	*Metabolites*	*Reactions*
***E*. *coli* K-12 (iJO1366)**	1807	2585	313	432
***S*. *enterica* LT2 (iRR1083)**	1803	2547	224	346
***P*. *putida* KT2440 (iJN746)**	917	1053	206	259
***P*. *stutzeri* A1501 (iPB890)**	1056	1135	113	173

#### Comparison of IndiMeSH with a population-based spatiotemporal metabolic network model

We compared IndiMeSH with COMETS (Computation of Microbial Ecosystems in Time and Space), a model designed primarily to investigate the growth and interrelation of bacterial communities in space using FBA and population-based approaches. In comparing IndiMeSH with COMETS predictions, we sought to verify the potential of IndiMeSH in simulating trophic dependencies of multispecies bacterial communities without making prior assumptions. For this reason, simulations were generated mimicking the two-member consortium investigated in the original publication [21]. This consortium consisted of two mutant strains, *E*. *coli* K-12 deficient in methionine production and *S*. *enterica* LT2. *E*. *coli* relies on the production of methionine by *S*. *enterica*, whereas *S*. *enterica* is dependent on the release of acetate by *E*. *coli* in microaerobic conditions due to its inability to directly metabolize lactose, thus engaging in a mutualistic relationship where neither species can grow in the absence of the other [40]. S. enterica has been genetically engineered to produce methionine which is incorporated in the metabolic network by linking the excretion of methionine to the biomass function. A summary of boundary conditions for all metabolic networks can be found in S1 Table in the supplementary material.

To simulate microbial life on a two-dimensional surface in IndiMeSH, a rectangular, saturated pore network (coordination number: 4) was created with the same topology as in the COMETS simulations (for details see S1A Fig in the supplementary material). Each pore has dimensions of 500x250x70 microns (LxWxH). The width of the pores was reduced (i.e. not a square patch as in COMETS) to conserve the total surface area (taking into account overlapping corners of the pores). Pore depth was calculated based on the characteristic diffusion length within one time step. Lactose and oxygen were supplemented at concentrations of 2.92 mM and 0.1 mM, respectively. Acetate and methionine are solely produced through bacterial metabolism and do not have any external sources. A fixed nutrient diffusion coefficient in water of 5*10^−10^ m^2^s^-1^ is used for all simulations [21]. Since the simulation represents bacterial colonies growing on agar, the population was represented using “super agents” in IndiMeSH, each representing 100 identical bacterial cells with synchronized mobility. An inoculum of 3*10^−7^ g bacterial biomass thus contains 3000 super agents (assuming a cell mass of 10^−15^ kg [41]). 100 randomly chosen nodes were inoculated with 3000 super agents with prescribed *E*. *coli* to *S*. *enterica* ratios of 99:1 or 1:99, resulting in an initial community of 300,000 super agents. The total simulated time was 48 hours using 10 second time step. This time step is much shorter than reported in the COMETS simulation (6 minutes) and was selected to maintain the functionality of bacterial motility as a pseudo run-and-tumble mechanism. To simulate colony growth and expansion on agar surfaces, bacterial cell velocity was reduced to 2.2*10^−9^ ms^-1^ to obtain good agreement between COMETS biomass diffusion and IndiMeSH run-and-tumble biomass dispersal (S3 Fig in the supplementary material).

#### Comparison to previously published experimental data of bacterial spatial organization

A similar core IBM (without the FBA component) has been used in previous studies to investigate an experimental system designed to visualize and quantify bacterial spatial organization in pore networks [13]. The experimental system consisted of micrometric pore networks where boundary conditions could be controlled mimicking conditions found in soil aggregates. Two bacterial species, an obligate aerobe and facultative anaerobes, were used to elucidate the spatial organization and coexistence in response to carbon and oxygen counter gradients. The primary disadvantage of the previous mathematical model has been the representation of *P*. *veronii* 1YdBTEX2 as an obligate anaerobic species using simple Monod-type growth kinetics. In reality, *P*. *veronii* is capable of growing in both aerobic and anaerobic environments by adapting its metabolism. The lack of metabolic flexibility in the Monod-based application resulted in a certain discrepancy between experimental and simulation results, especially for the cases where facultative anaerobes were growing closer to the oxygen source [13]. In this study, we repeat these simulations using the reduced-scale metabolic networks in the IndiMeSH model with the same physical, hydrological and chemical domain setup. The physical domain comprised a fully connected hexagonal lattice (coordination number: 6) with each pore having a cuboid shape of 200x40x15 microns (LxWxH) and a total domain size of 1 cm by 1 cm. Four nutrients were included in the simulation: citrate (central source, 10 mM), nitrate (central source, 10 mM), oxygen (peripheral source, 0.27 mM) and acetate (produced anaerobically by bacterial cells, no physical source). For *P*. *putida* KT2440, a reduced core model of the genome-scale metabolic network iJN746 [42] was implemented. In the absence of a curated metabolic network for the *P*. *veronii* 1YdBTEX2, we have used a reduced-scale model of *P*. *stutzeri* A1501 (iPB890) [43] due to the similarity especially in facultative anaerobic capabilities of using denitrification under anoxic conditions. Eight realizations with varying inoculation numbers and ratios were used congruent to the original publication. A summary of the simulation setup is shown in S2B Fig in the supplementary material.

#### Glucose perfusion in soil aggregates

A main advantage of the IndiMeSH model is its capability to capture the aqueous phase configuration and related limitations to bacterial dispersal and nutrient diffusion landscape. Previously conducted experiments performed in 2mm soil aggregates investigated the effect of carbon perfusion by adding glucose after a 7 day incubation period as reported in [24]. These experiments were replicated within the IndiMeSH framework to describe and quantify the observed shift in bacterial total abundance due to the carbon perturbation. Boundary conditions of the simulations attempted to mimic the experimental conditions, although unknowns concerning the initial bacterial cell distribution and numbers remain, and the distribution and amount of carbon during the pre-incubation as well as the matric potential during glucose addition were not reported. Congruent to the experimental system, simulations were performed in 2mm diameter pore networks representing soil aggregate cross-sections. The approximate pore size distribution was taken from the experimental data (lognormal distribution with mean pore diameter of 10 microns and variance 5 microns). An initially homogeneously inoculated bacterial community was grown into stationary phase for 7 days after which a pulse of glucose (333 mM) was added to the simulations. The matric potential was kept at -10 kPa. During the 7 day incubation period, a central glucose and nitrate source was present (constant concentration of 0.5 mM and 0.4 mM, respectively). Oxygen was provided from the periphery which penetrated the aggregate completely due to the unsaturated conditions. No analysis concerning the species was performed in the experiment. For the simulations, a reduced scale metabolic network of P. stutzeri A1501 was chosen (iPB890) where 1000 cells were inoculated homogeneously across the pore network. Total simulation time was 9 days at a 10 s time step with glucose added after 7 days.

#### Contrasting hydration conditions with varying pore size distributions

The previous case studies included scenarios with saturated (spatial self-organization) and unsaturated (glucose perfusion) conditions. However, a strategic comparison of simulations under wet and dry conditions in the same pore network is missing. For this reason, a final case study containing three pore networks (large, medium and small pores) and two hydration levels (-1 kPa for wet and -10 kPa for dry conditions) was created. The simulation domain comprised of a 4 mm diameter carbon hotspot representing a soil aggregate or rhizosphere cross-section. The pore lengths was adapted for each network to conserve porosity. Pore size distributions were taken from a lognormal distribution with mean radius of 5, 50 and 200 microns for the small, medium and large pore networks, respectively, with corresponding standard deviations of 5, 10 and 50 microns. Simulations included central glucose and nitrate sources with a peripheral oxygen source (0.27 mM). Glucose and nitrate concentrations were adapted for each network to represent constant flux boundary conditions. Acetate was included in the simulations as an intermediate metabolite without any physical sources. Pseudomonas stutzeri A1501 was used as a model organism (reduced metabolic network iPB890) due to its facultative anaerobic growth capability. The total simulated time was five days with a 10 s time step.

## Results

### Evaluation of the GEM and rGEM

The performances of genome-scale metabolic networks (GEM) and their reduced-scale networks (rGEM) were compared using flux variability analysis in varying environmental conditions. Table 2 summarizes the median percentage and standard deviation of common reactions that differ to varying degrees in their maximum or minimum flux between the GEM and rGEM models for all environmental conditions. Overall, the performance of the reduced-scale model was very similar to the genome-scale models, with approximately 85% of all reactions within the models having minimal flux and maximal flux differences of less than 1 mmol/gDW/h. In addition, the standard deviations were small for most metabolic network comparisons, with the exception of the *E*. *coli* metabolic networks. S4 Fig in the supplementary material relates the comparison of the metabolic networks to environmental conditions to elucidate potential biases due to specific combinations of nutrient uptake rates. The metabolic networks of *E*. *coli* perform differently at the combination of high lactose, low methionine and medium to high oxygen uptakes.

**Table 2:** Evaluation of metabolic network reduction and comparability to genome-scale metabolic networks. Genome-scale and reduced-scale metabolic networks were compared using flux variability analysis whilst exposed to different environmental conditions. A percentage of the maximum uptake rate (20%, 60% and 100%) for each nutrient was cross-combined with all other nutrient levels in order to generate 81 conditions in the case of four nutrients and 27 conditions in the case of three nutrients. Presented is the median percentage of all reactions having a minimal flux or maximum flux difference falling within the respective category (with standard deviation in brackets).

	<0.1 mol/gDW/h	> 0.1 mmol/gDW/h < 1 mmol/gDW/h	> 1 mmol/gDW/h < 10 mmol/gDW/h	> 10 mmol/gDW/h
***E*. *coli* K-12 (iJO1366)**	0.75 (0.16)	0.07 (0.02)	0.08 (0.13)	0.08 (0.08)
***S*. *enterica* LT2 (iRR1083)**	0.92 (0.03)	0.05 (0.03)	0.00 (0.01)	0.04 (0.01)
***P*. *putida* KT2440 (iJN746)**	0.77 (0.02)	0.11 (0.05)	0.10 (0.06)	0.02 (0.01)
***P*. *stutzeri* A1501 (iPB890)**	0.82 (0.07)	0.06 (0.07)	0.05 (0.02)	0.04 (0.03)

### IndiMeSH vs. COMETS predictions

**Fig 2:**
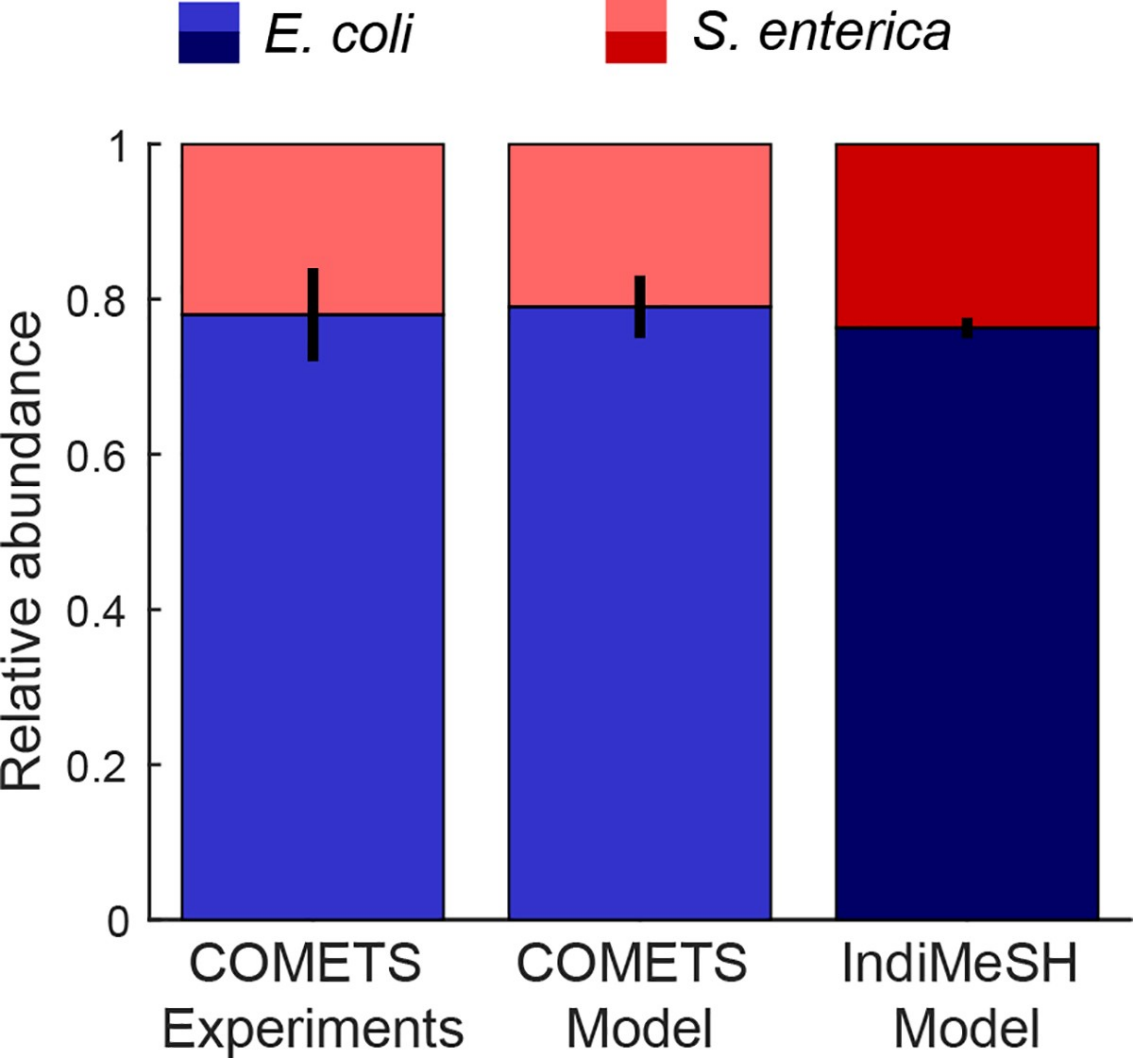
Relative abundance of *E*. *coli* and *S*. *enterica* in the IndiMeSH simulations and original COMETS simulations and experimental results. Combined relative abundance of initial inoculation ratios (99:1 and 1:99 of *E*. *coli S*. *enterica*, three replicates each, inoculated at 100 random nodes) for experimental results, COMETS simulations and IndiMeSH simulations after 48h of growth. Error bars represent one standard deviation.

- The aim of comparing COMETS with IndiMeSH was to verify IndiMeSH ability to predict bacterial community trophic interactions in the absence of prior assumptions regarding nutrient uptake (in contrast with Monod kinetic models). Fig 2 shows the final ratio of *E*. *coli* and *S*. *enterica* after 48 h of simulated growth, including the experimental results and model simulations from the original COMETS publication [21]. Both inoculation ratios (99:1 and 1:99 as *E*.*coli*:*S*.*enterica* inoculated at 100 randomly chosen nodes) were included, demonstrating the metabolic interdependence of the two species and the convergence of the system to the same stable community composition. No significant difference was found between IndiMeSH simulations and both COMETS experimental and simulation results (p value of 0.66 and 0.33, respectively, two-tailed t-test). Additional simulations were performed to ensure that the motility algorithm of IndiMeSH and changing to super agents resulted in similar biomass dispersion characteristics as in the diffusion algorithm used in COMETS. S3 Fig in the supplementary material shows the biomass dispersion for the COMETS algorithm and IndiMeSH run-and-tumble algorithm, both with and without super agents.

### Spatial organization using IndiMeSH

**Fig 3:**
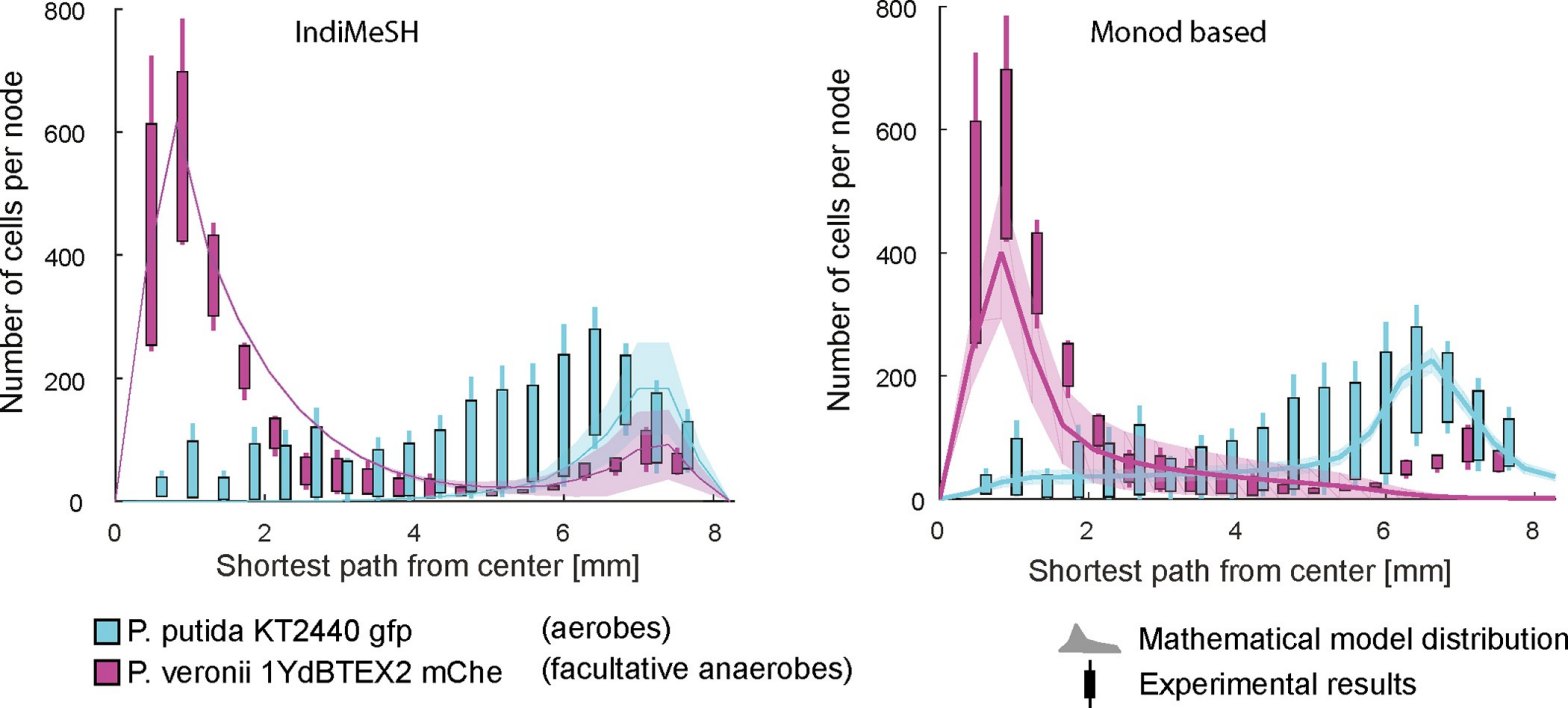
Distribution of obligate aerobic *P*. *putida* (cyan) and facultative anaerobic *P*. *veronii* (magenta) along the carbon-oxygen counter gradient from the central port to the peripheral ports. Combined experimental results (boxplots, whiskers indicate minimum and maximum of data) and simulation results (shaded area with 95% of all cells, thick line represents mean) in a well connected, artificial pore network using IndiMeSH (left) and Monod parametrization (right). Segregation of facultative anaerobes into an anaerobically growing (central port, anoxic) and peripheral, aerobically growing subpopulation can be observed in the results using IndiMeSH which is absent in the results using Monod parametrization. The observed shift of obligate aerobes towards the periphery is due to the chosen canonical half saturation coefficient in IndiMeSH (0.05 mM) compared to the Monod half saturation coefficient in the original model (0.0063 mM).

To evaluate the ability of IndiMeSH predict the spatial organization of a bacterial community in pore networks due to nutrient cross-gradients (where different metabolism is required), we compared IndiMeSH predictions with previously published experimental data [13]. Fig 3 depicts the resulting spatial distribution of *P*. *putida* (further termed aerobes) and *P*. *veronii/P*. *stutzeri* (further termed facultative anaerobes) along the shortest path from the central to the peripheral ports of the physical and simulated pore networks (details of the physical domain and chemical boundary conditions are described in the methods and shown in S2 Fig of the supplementary material). The aerobes proliferate closer to the periphery than the facultative anaerobes, with a small subpopulation of facultative anaerobes residing at the oxygen-rich peripheral ports. The magnitude of the populations of both species is captured well both at the central and peripheral ports, although the location of aerobes is predicted to be in closer proximity to the peripheral port than observed experimentally. For the facultative anaerobes, the simulations capture both the central population utilizing anaerobic metabolism (fermentation) as well as the peripheral sub-population using aerobic respiration.

### Metabolic adaptation to rapid changes

**Fig 4:**
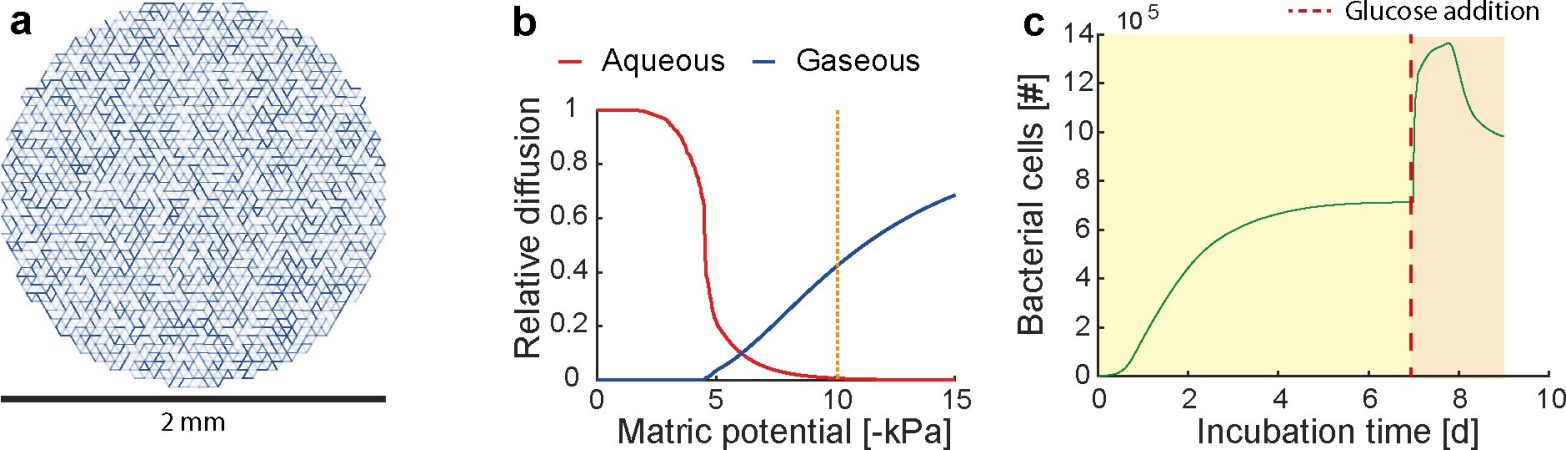
Response of bacterial population to a shift in boundary conditions represented as glucose perfusion into a soil aggregate cross-section. (a) The pore network used for the simulation congruent to the soil aggregates used in the experiment. The distribution of glucose within the pore network is visualized prior to glucose perfusion. (b) Relative diffusion compared to a saturated pore network is calculated using the Millington-Quirk equation. The dashed orange line indicates the state of the pore network at the matric potential set experimentally and within the simulations, limiting nutrient diffusion in the aqueous phase whilst facilitating gaseous diffusion due to unsaturated pores. (c) Growth curve of the bacterial population showing a secondary growth phase after glucose addition with a decline in population size approaching the previously established stable population size due to the system carrying capacity.

We evaluated the capability of IndiMeSH to predict the shift in total bacterial population size due to perturbations in boundary conditions in the form of glucose perfusion into soil aggregates previously under certain steady ecological state. Simulations were set up to mimic experiments performed on 2 mm soil aggregates where the bacterial community was first grown into steady state for 7 days after which glucose was added at high concentrations to observe the short-term impact on the function and the spatial distribution of soil bacteria and fungi [24]. Fig 4 depicts the pore network employed for the simulation congruent to the reported pore size distribution in the experiment (Fig 4A), the resulting diffusional scheme of the pore network calculated using Millington-Quirk equation (Fig 4B) as well as the total temporal response of the community during the pre-incubation and dynamic response to the addition of a glucose pulse (Fig 4C). Due to the unsaturated conditions (expressed by a matric potential of -10 kPa), the diffusion of nutrients confined to the aqueous phase was severely limited whereas oxygen in the gas phase was able to penetrate the pore network rapidly. The primary mode of growth was thus aerobic respiration within all regions of the pore network. During the pre-incubation period, the bacterial population grew into steady state dictated by the carrying capacity of the supplied carbon. Glucose addition after 7 days triggered a secondary rapid growth phase until a maximum is reached after approximately 8 days into the simulation. The population then starts to decline due to the lack of carbon availability, tending towards the previously established population size due to the system carrying capacity. Glucose perfusion does not have a homogeneous effect on the population concerning space. The relative increase in population size of the outer aggregate faction (peeling off 1 mm of the aggregate conceptually) compared to the inner faction is 2.5 to 1.1, respectively. This is similar to the observation made within the experiments where they reported a total increase in cell number of 1.6 and 1.3 for the outer and inner faction, respectively. Within the simulation this is mostly due to the availability of carbon at the periphery which otherwise is intercepted by the population closer to the center of the aggregate. Thus, the initial spatial distribution of the community was disrupted by the change in carbon availability, creating a more heterogeneous distribution of the population.

### Contrasting hydration conditions with varying pore size distributions

**Fig 5:**
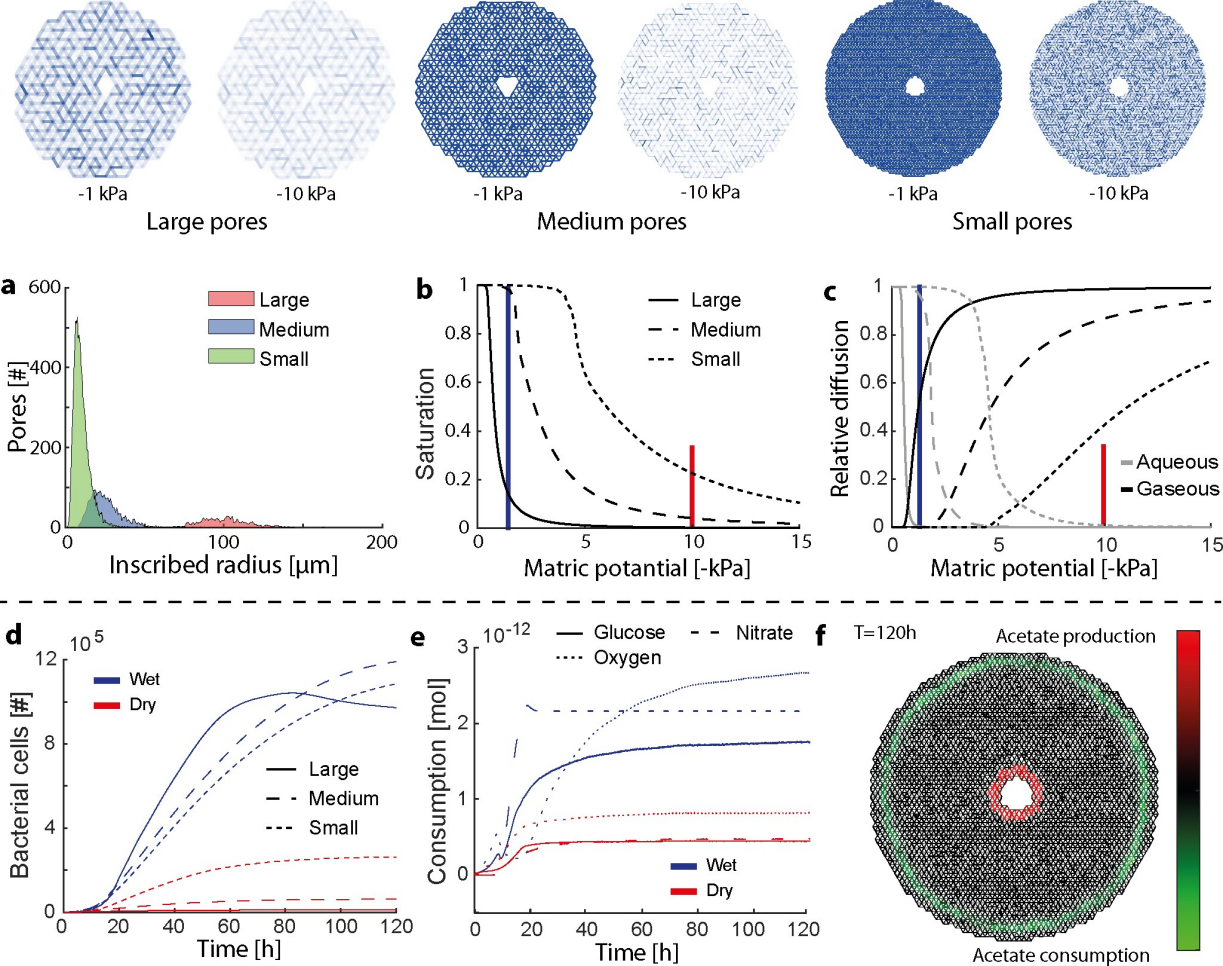
Strategic analysis of pore size distribution and hydration conditions. Visualization of the degree of connectivity for all three pore networks with varying mean pore size at two hydration conditions. (a) Histogram of pore sizes taken from lognormal distributions with varying mean and standard deviations. (b) Resulting soil water characteristic curve including indications of matric potential taken as wet (-1 kPa) and dry (-10 kPa) conditions. (c) Calculated relative diffusion for all pore networks using the Milling-Quirk equation. (d) Bacterial growth dynamics depending on pore network and hydration condition for the whole simulation time of 120 h. (e) Bacterial consumption for the small pore size network and varying hydration conditions. (f) Spatial distribution of acetate production and consumption. Acetate is produced at the carbon rich center due to overflow metabolism (electron acceptor limited) and consumed at the periphery via aerobic respiration.

Finally, we created simulations with two contrasting hydration conditions in three different pore networks to highlight the capability of IndiMeSH to capture and predict the shift in metabolic activity due to the interplay of soil physics, aqueous configuration and bacterial metabolism. Pore size distributions resembling different soils are shown in Fig 5A with the related soil water characteristic curves shown in Fig 5B. A matric potential of -1 kPa and -10 kPa, was applied to simulate wet and dry conditions, respectively, with profound consequences for the hydration conditions for each network as indicated by the green lines in Fig 5B. The degree of saturation in turn dictates the relative diffusion of gaseous and aqueous nutrients as shown in Fig 5C. In dry conditions, aqueous nutrients are restricted in their diffusive capability due to thin water films whilst gaseous nutrient diffusion (e.g. oxygen) is facilitated. Due to the constant flux boundary conditions, bacterial populations reach similar magnitudes in wet conditions as shown in Fig 5D. Under dry conditions, diffusion of substrate in the large pore network is restricted more profoundly compared to the small pore networks, which results in a lower total carrying capacity. A main difference between the saturated and unsaturated simulation also lies in the metabolic activity of the population. Due to the penetration of oxygen deep into the aggregate for all pore sizes under dry conditions, the sole mode of growth is via aerobic respiration as shown in the consumption pattern for the small sized pore domain in Fig 5E. Under wet conditions, the additional consumption of nitrate indicates the use of denitrification pathways. Under these conditions, overflow metabolism occurs at the center due to an imbalance in electron donor and acceptor ratio resulting in the production of acetate, which is consumed at the periphery using aerobic respiration as shown in Fig 5F.

## Discussion

The IndiMeSH model has been developed to capture the multifaceted bacterial metabolism of individual-based agents inhabiting a complex habitat such as soil. The use of adaptive metabolic networks enables a life-like transition between aerobic and anaerobic conditions compared to empirical and arbitrary kinetic models. Additionally, FBA predicts uptake and secretion rates depending on locally variable nutrient conditions and thus enables simulations of trophically interacting bacterial species within a complex medium such as, but not limited to, soil. The heavy computational burden related to the motility of individual bacterial cells and the numerous iterations of the FBA requires the reduction of genome-scale metabolic networks using the previously published redGEM [36] and lumpGEM [37] algorithms. The implementation of reduced-scale metabolic models (rGEM) significantly reduced the computational time whilst retaining the salient features and predictability offered by the genome-scale models (GEM) (Table 1). Although such a reduction (GEM to rGEM) may not be crucial for very small physical domains, simulations of ecologically relevant systems where gradients of environmental variables persist would require spatial domains with a few thousand nodes. For example, in the micromodel comparison (Fig 3) we considered approximately 3000 nodes and two species, thus solving 6000 FBA problems per time step; hence, the use of rGEM greatly improved computational efficiency. Compared to other individual based metabolic models the primary benefit of reduced models is not due to the physical domain size (e.g. BacArena simulates domains containing 100 by 100 grid nodes [22]), but the small time step dictated by the motility algorithm and related large number of iterations in total. Except for *E*. *coli*, all reduced-scale models perform very similarly to their genome-scale counterparts. Reactions which have a net flux difference greater than 10 mmol/gDw/h are predominantly attributed to internal cycles within the genome-scale models or cycling of nutrients between compartments of the genome-scale model that are absent in the reduced-scale model, as has been previously reported [39]. There is a discrepancy in acetate metabolism between the reduced-scale and genome-scale models of *E*. *coli*. This is due to overoptimistic predictions of the genome-scale model where the predicted high fluxes can only be achieved through net CO_2_ fixation, which is unlikely [39].

The main difference between a group of spatiotemporal metabolic models and the newly developed IndiMeSH lies in the ability of the latter to represent complex habitats such as soil, including pore spaces, aqueous phase configurations and related constraints to chemical diffusion and bacterial dispersion. The IndiMeSH model is designed for a different range of applications than the COMETS model; however, we obtained comparable simulation scenarios through simple adjustments to the physical and biological domain of the model. These include the representation of bacterial cells as super agents as well as a reduction in velocity to account for growth on agar. The use of super agents has been implemented in the past [44,45] and in other studies have been shown to have limited effects on simulation results as long as the scaling ratio is sufficiently small [46]. Using these modifications, IndiMeSH accurately reproduced the COMETS results. These simulations rely on an appropriate reduction of the genome-scale models preserving the stoichiometric relations between consumed and excreted nutrients and demonstrate the ability of IndiMeSh to replicate and predict bacterial growth based on trophic interactions, a process ubiquitous in dense bacterial hotspots. Since both COMETS and IndiMeSH are essentially two-dimensional simulations, the oxygen concentration was kept artificially low to imitate conditions experienced by the layer of cells close to the agar itself. In reality, a bacterial colony growing on agar is a dynamic system where oxygen is consumed at the periphery, resulting in multiple micro niches and redox conditions within the colony itself [47]. Individual-based models coupled with metabolic networks focusing on colony growth are able to capture this phenomenon well [23], however the scale of those models restricts simulations of a large domain with hundreds of colonies.

The importance of emergent spatial patterns and bacterial spatial organization prompted us to examine how IndiMeSH reproduces previously published experimental data of bacterial distributions in micrometric pore networks dominated by carbon-oxygen counter gradients [13]. The original IBM represented facultative anaerobes as obligate anaerobes, which resulted in consistent discrepancies between model and experimental results especially for facultative anaerobes proliferating close to the oxygenated peripheral ports. Integration of metabolic networks as a means of growth calculation enabled us to capture the segregation of facultative anaerobes into a denitrifying population close to the carbon source and a subpopulation using aerobic respiration (Fig 3). Despite possibilities to represent facultative anaerobes using Monod kinetics and an arbitrary oxygen-threshold-switching strategy (e.g. [15]), IndiMeSH managed to capture the facultative behavior more naturally without an artificial construct of bimodal metabolism. Originally, the interaction between the two species suggested an indirect commensalism where the anaerobes were required to release sufficient carbon to the periphery for complete oxygen consumption by the aerobes and provision of an anoxic niche. Through the integration of metabolic networks and additional intermediate metabolites in the form of acetate (to ensure mass balance of carbon within the simulation), the cooperation between the species was shown to be a passive interaction where acetate was produced due to a lack of electron acceptor (not sufficient nitrate available) at the central port. Diffusion of acetate to the periphery enabled the aerobic community (both facultative anaerobes and obligate aerobes) to grow closer to the oxygen-rich peripheral port. This mechanism is less susceptible to perturbations and provides a more plausible explanation for cooperation compared to the scenario where one species “farms” the other.

Abrupt perturbations are ubiquitous in all natural systems such as soil, either due to rapid changes in hydration conditions following rainfall or irrigation, or chemical alteration by the application of fertilizer or contaminants to the soil, or physical alterations such as tillage or rapid soil compaction. To test the capability of IndiMeSH to simulate such dynamic changes in environmental conditions, a simulation scenario was created where a change in electron donor availability alters the macroscopic conditions within a soil aggregate cross-section based on experimental results of glucose perfusion in soil aggregates [24]. These experimental results describe a non-uniform effect in space as evidenced from peeling off 1 mm of the aggregate with 1.6 fold increase in bacterial total abundance in the periphery relative to 1.3 in the core (in the numerical experiment we observed 2.5 fold outer to 1.1 inner). The interpretation we provide to these interesting experimental results is that the perfusion with glucose alleviates the carbon flux limitations in natural aggregates, which is controlled by the inner (largely anaerobic) population. The perfusion creates a situation where carbon and oxygen become available at the periphery and promote the rapid growth during the availability window (until the carbon pulse is consumed). In the simulation, the total population size starts to decline after peaking around 8 days into the simulation. This is not easily verified in the experimental data, but acknowledging that only a fraction of the total population was reported to be dividing (approximately 4.5% to 8.2% of the total population [24]), assuming that the population has reached steady state or is declining does not seem farfetched. These simulations represent a scenario where the physical representation of soil in the form of angular pore networks plays a crucial in shaping the diffusional landscape of the microbial habitat and overall population growth–a unique feature of the presented model framework.

An important aspect differentiating IndiMeSH from other current spatiotemporal metabolic network models is the accuracy with which the physical habitat of the bacterial cells is represented. Angular pore networks enable to link the aqueous phase configuration dictated by the pore geometry to the underlying chemical conditions and ultimately to the spatial landscape of bacterial metabolism. For the same matric potential, pore networks of different mean diameter expose bacteria to fundamentally different growth conditions as shown for instance in Fig 5B at -1 kPa. Both the medium and small pore networks are saturated (nearly 100%) at this matric potential whereas the pore network containing large pores is desaturated with profound consequences for oxygen diffusion dynamics and bacterial motility. Potentially more important is the ability of IndiMeSH to capture the change in aqueous phase configuration for the same pore network at varying matric potentials. As shown in Fig 5E, the change in matric potential not only alters the magnitude of nutrient consumption but also the relative ratio of for instance glucose to nitrate consumption. In wet conditions, the community is split into two distinct sub populations with one growing anaerobically via denitrification and producing acetate at the center and a second which grows via aerobic respiration of acetate at the periphery of the domain (Fig 5F). In dry conditions, aerobic respiration is favored due to the increased gaseous diffusion at -10 kPa (Fig 5C). The difference in population size depending on hydration conditions is due to the reduction in carbon diffusion under dry conditions. It is important to note that these contrasting conditions emerge due to a single change in model input files–the matric potential–which induces substantial changes to both the physicochemical aspects of the model as well as bacterial growth strategy. Hence, the physical description of the pore habitat, related diffusional constraints and bacterial self-organization in complex pore systems are features that distinguish IndiMeSH from other current spatiotemporal metabolic models such as COMETS[21] or BacArena[22].

IndiMeSH offers a representation of metabolic versatility with an individual-based model of bacterial life in soil habitats. Metabolic networks enabled a natural transition between aerobic and anaerobic pathways and associated nutrient consumption patterns, inducing trophic interactions without prior assumptions, as required by mathematical models using simple growth kinetics. The detailed description of the physical habitat and aqueous phase and emergent nutrient diffusion fields give rise to complex metabolic landscapes with changes in metabolic activity at the millimeter scale. Due to the modular design of the IndiMeSH model, individual components can be modified to represent, for instance, a different physical habitat, method of motility or metabolic network optimization (thermodynamic FBA, FBA with molecular crowding). Thus, the applicability of the model is endless, ranging from the quantification of bacterial metabolism in soil, or the growth of bacteria during food spoilage, to the proliferation of Pseudomonas aeruginosa in the lungs of cystic fibrosis patients.

## Supporting information

S1 Fig Geometry definition for angular pore cross-sections. The geometry of each triangular pore in the pore network is defined by the inscribed circle radius r and central angle γ. Rectangular pore geometry is determined by the width W and height H for each pore. Interfacial curvature R (depending on matric potential Ψ) determines the total water filled cross-sectional area A_w_. If Aw is smaller than the total pore cross-sectional area and has a continuous unsaturated connection to an air interface, the pore is considered to be unsaturated. Finally, the water film thickness WFT, which dictates bacterial swimming velocity, is defined as the radius of an inscribed circle between the pore walls and the liquid-air interface.
(TIF)

S2 Fig Habitat geometry, boundary conditions and inoculation location for all simulation scenarios. (a) The COMETS habitat geometry contains a rectangular grid consisting of individual rectangular pores with homogeneous size (L x W x H being 500, 250 and 70 microns, respectively). Constant concentration boundaries of 2.92 mM lactose and 0.10 mM oxygen are supplied homogeneously throughout the domain. Acetate and methionine are solely produced by bacterial metabolism. 100 randomly chosen nodes are inoculated with 3000 super agents each. (b) For the micromodel comparison, a hexagonal lattice of individual cuboid pores (L x W x H being 200, 40 and 15 microns, respectively) is used with peripheral oxygen sources (0.27 mM) and central citrate and nitrate sources (each 10 mM). All bacterial cells are inoculated at the center. (c) For the glucose perfusion scenario, a hexagonal lattice with individual triangular pores (100 micron length, varying widths and heights as described in the Methods) is used with peripheral oxygen source (0.27 mM) and central glucose and nitrate sources (0.5 and 0.4 mM, respectively). After 7 days, an additional pulse of glucose was added, homogeneously distributing 333 mM of glucose in all pores. 1000 cells were inoculated randomly across the domain.
(TIF)

S3 Fig Comparison of COMETS and IndiMeSH motility predictions. Algorithms were compared in a one-dimensional pore (2000 microns length with 100 nodes) and inoculation of 3*10^-7^g bacterial biomass at the center node. IndiMeSH predictions were produced both for individual bacterial cells and super agents with a scaling ratio of 100 (i.e. one supercell contains 100 individual bacterial cells). Predictions of the two models are congruent with a velocity of 8 microns per hour for bacterial swimming motility in the IndiMeSH model.
(TIF)

S4 Fig Comparison of *E. coli* GEM and rGEM in varying environmental conditions. 81 environmental conditions were created by cross-combination of 20%, 60% and 100% of the maximum uptake rate for all four nutrients contained in the COMETS simulation. Color indicates the percentage of common reactions in the two models that differ in carrying flux less than 1 mmol/gDW/h. A discrepancy in metabolism is visible in high lactose and oxygen conditions depending on acetate availability. This is due to an overoptimistic prediction of the *E*. *coli* genome-scale network (see discussion for details).
(TIF)

S1 Table Summary of maximum uptake rates imposed for all metabolic networks and simulation scenarios. If sufficient nutrient is available, cells consume nutrients at the maximum rate. At low nutrient concentrations, the amount is divided equally between all cells residing within a node, resulting in uptake rates lower than the maximum uptake rate.
(DOCX)

S2 Table List of parameters that can be set by the model user. This table contains a list of all parameters that can be set by the user for simulations containing multiple substrates and species. The list is exhaustive excluding the actual topology of the pore network (i.e. node coordinates, adjacency matrix and boundary location).
(DOCX)

S1 Data Mathematical model written in Matlab, metabolic networks and files containing simulation setup and boundary conditions for all case studies. (ZIP)
