# Supplementary figures and images for: Modeling metabolic networks of individual bacterial agents in heterogeneous and dynamic soil habitats (IndiMeSH)
Source: PLoS Comput Biol. 2019 Jun 19;15(6):e1007127. doi: 10.1371/journal.pcbi.1007127 (PMC6583959; doi:10.1371/journal.pcbi.1007127)

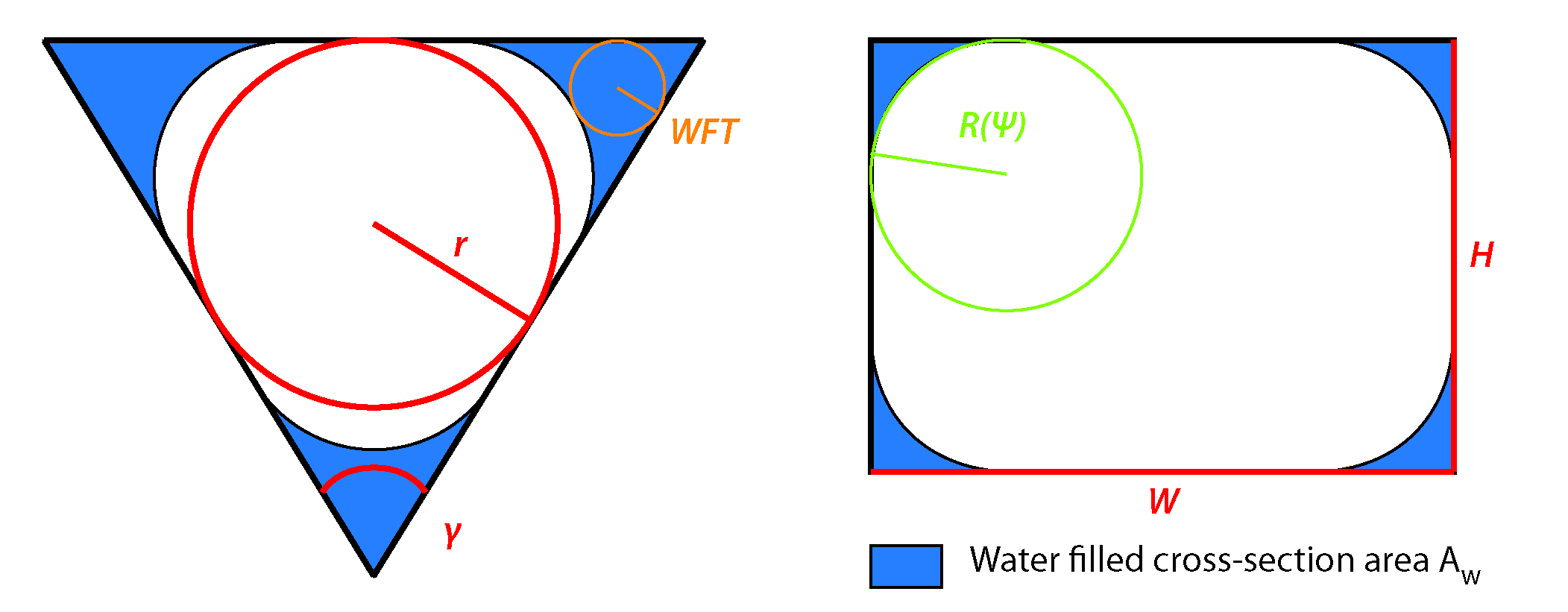

Supplement: S1 Fig — The geometry of each triangular pore in the pore network is defined by the inscribed circle radius r and central angle γ. Rectangular pore geometry is determined by the width W and height H for each pore. Interfacial curvature R (depending on matric potential Ψ) determines the total water filled cross-sectional area Aw. If Aw is smaller than the total pore cross-sectional area and has a continuous unsaturated connection to an air interface, the pore is considered to be unsaturated. Finally, the water film thickness WFT, which dictates bacterial swimming velocity, is defined as the radius of an inscribed circle between the pore walls and the liquid-air interface. (TIF) [file pcbi.1007127.s001.tif]

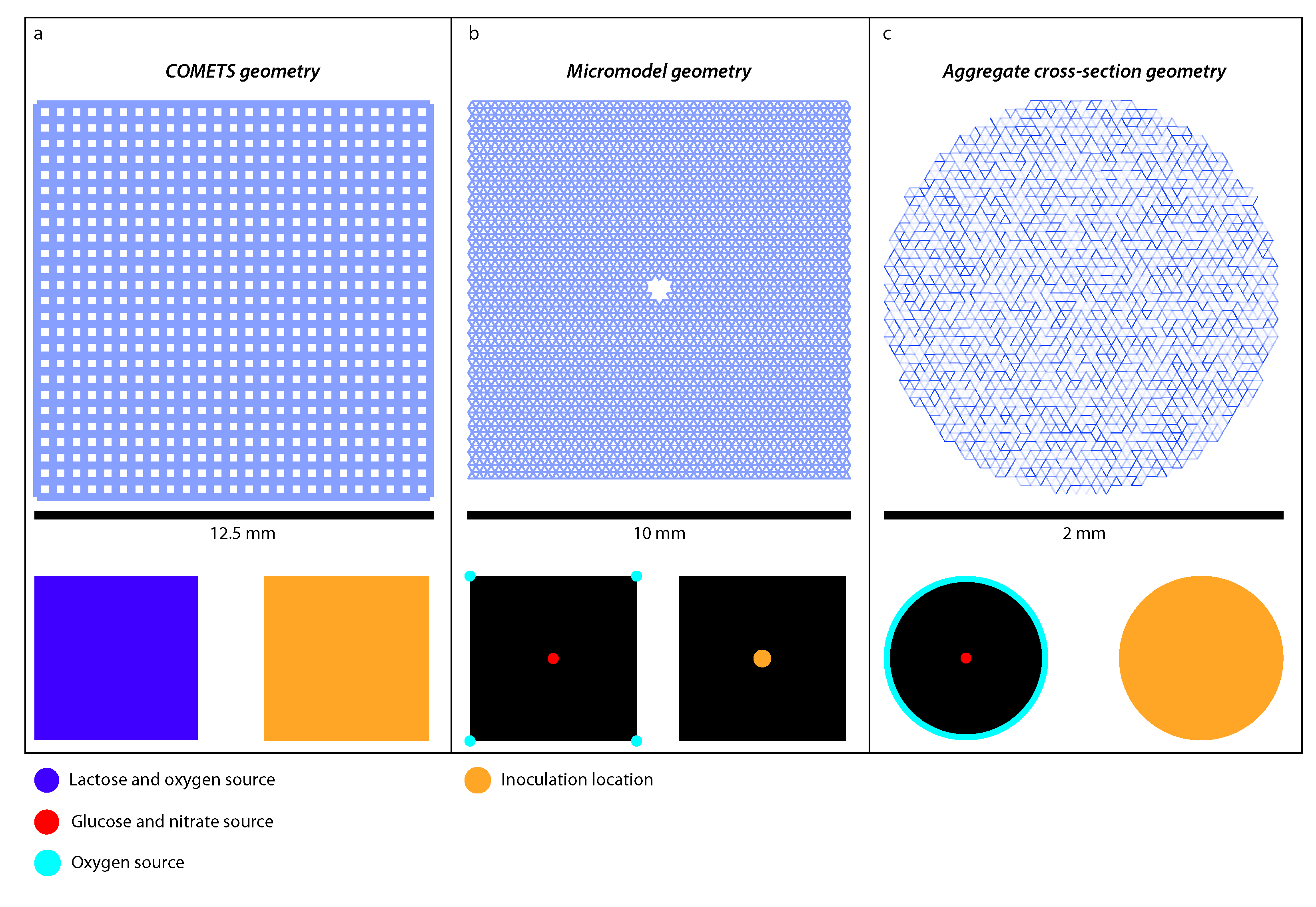

Supplement: S2 Fig — (a) The COMETS habitat geometry contains a rectangular grid consisting of individual rectangular pores with homogeneous size (L x W x H being 500, 250 and 70 microns, respectively). Constant concentration boundaries of 2.92 mM lactose and 0.10 mM oxygen are supplied homogeneously throughout the domain. Acetate and methionine are solely produced by bacterial metabolism. 100 randomly chosen nodes are inoculated with 3000 super agents each. (b) For the micromodel comparison, a hexagonal lattice of individual cuboid pores (L x W x H being 200, 40 and 15 microns, respectively) is used with peripheral oxygen sources (0.27 mM) and central citrate and nitrate sources (each 10 mM). All bacterial cells are inoculated at the center. (c) For the glucose perfusion scenario, a hexagonal lattice with individual triangular pores (100 micron length, varying widths and heights as described in the Methods) is used with peripheral oxygen source (0.27 mM) and central glucose and nitrate sources (0.5 and 0.4 mM, respectively). After 7 days, an additional pulse of glucose was added, homogeneously distributing 333 mM of glucose in all pores. 1000 cells were inoculated randomly across the domain. (TIF) [file pcbi.1007127.s002.tif]

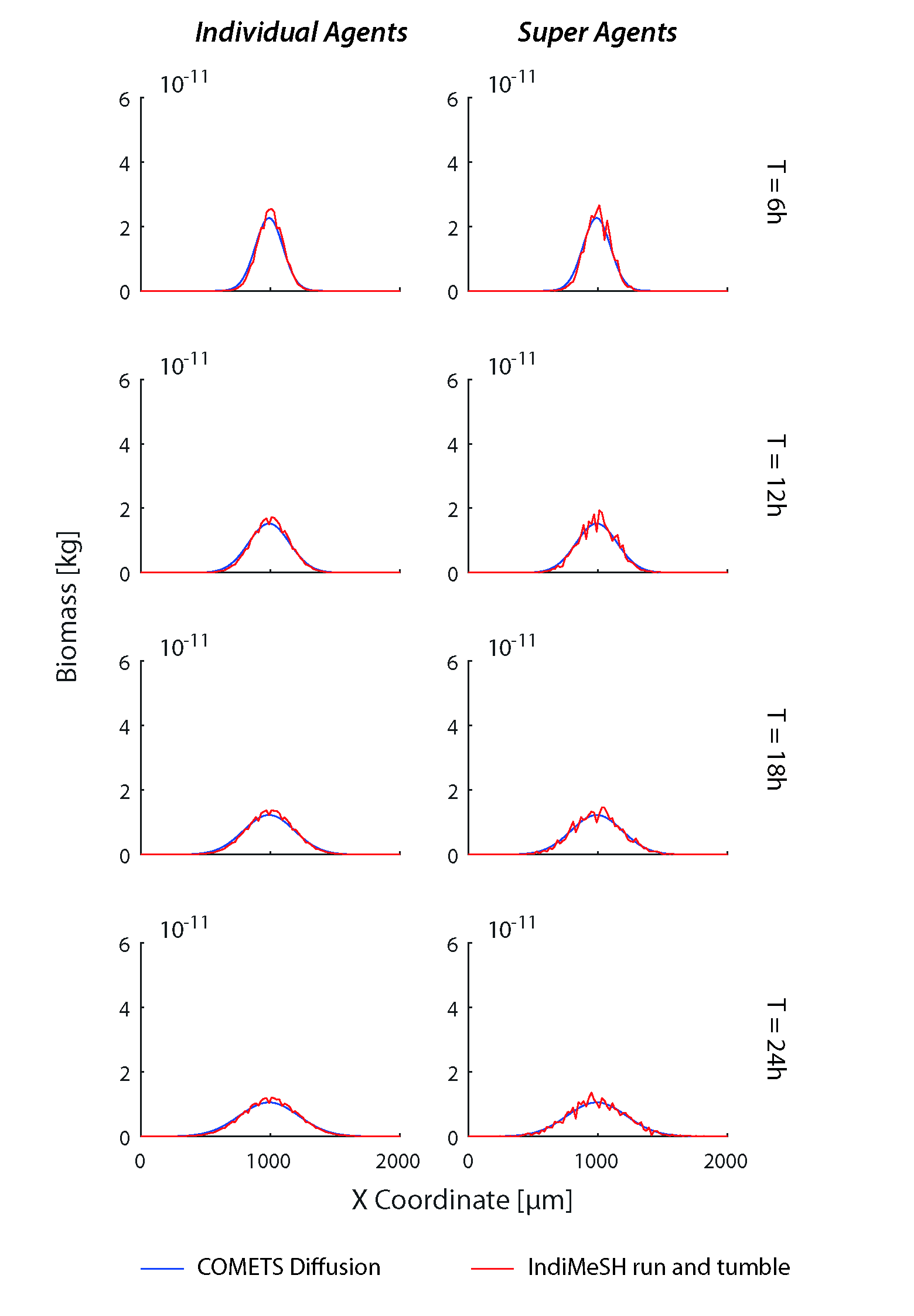

Supplement: S3 Fig — Algorithms were compared in a one-dimensional pore (2000 microns length with 100 nodes) and inoculation of 3*10-7g bacterial biomass at the center node. IndiMeSH predictions were produced both for individual bacterial cells and super agents with a scaling ratio of 100 (i.e. one supercell contains 100 individual bacterial cells). Predictions of the two models are congruent with a velocity of 8 microns per hour for bacterial swimming motility in the IndiMeSH model. (TIF) [file pcbi.1007127.s003.tif]

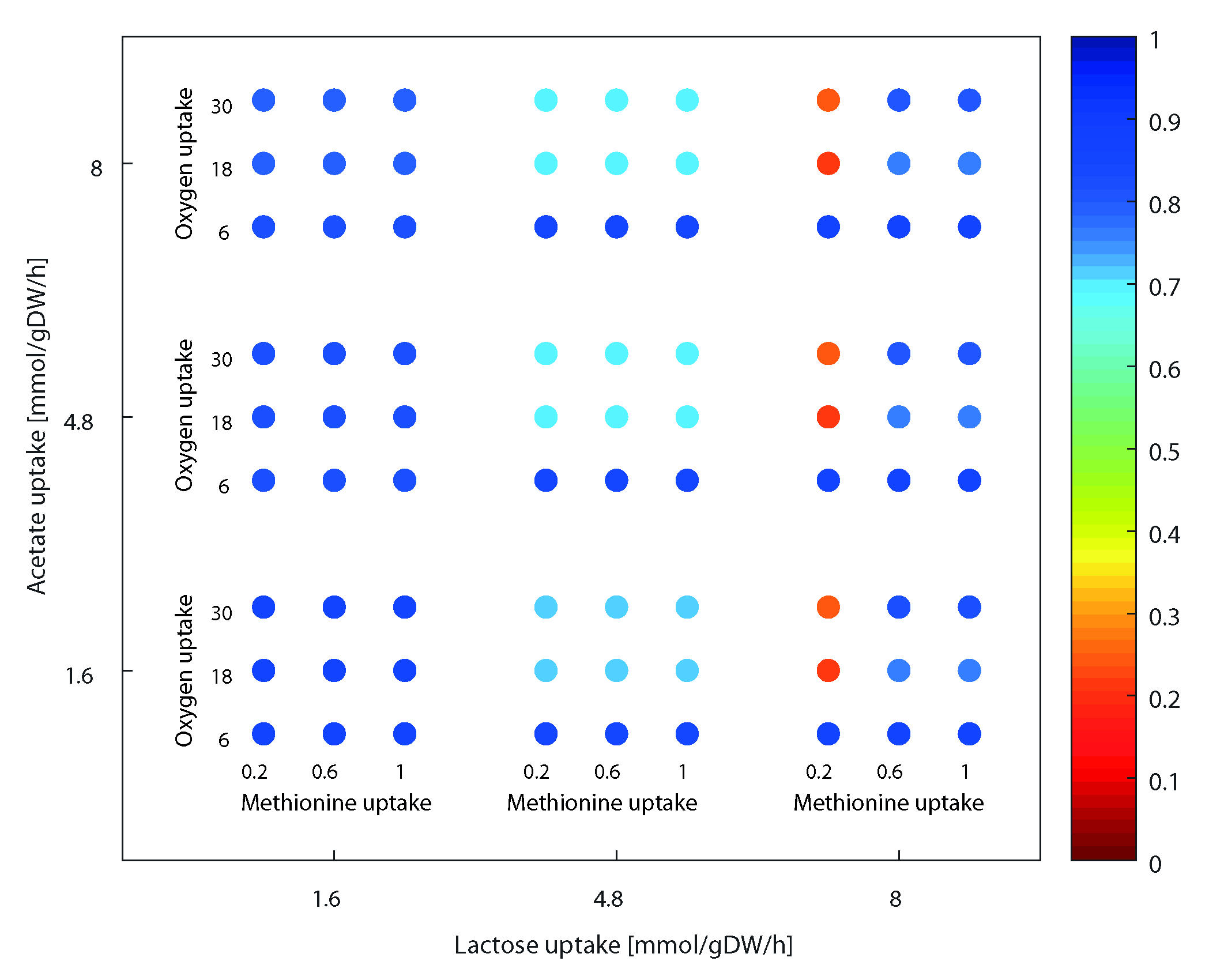

Supplement: S4 Fig — 81 environmental conditions were created by cross-combination of 20%, 60% and 100% of the maximum uptake rate for all four nutrients contained in the COMETS simulation. Color indicates the percentage of common reactions in the two models that differ in carrying flux less than 1 mmol/gDW/h. A discrepancy in metabolism is visible in high lactose and oxygen conditions depending on acetate availability. This is due to an overoptimistic prediction of the E. coli genome-scale network (see discussion for details). (TIF) [file pcbi.1007127.s004.tif]
