# Supplementary material for: Modeling metabolic networks of individual bacterial agents in heterogeneous and dynamic soil habitats (IndiMeSH)
Source: PLoS Comput Biol. 2019 Jun 19;15(6):e1007127. doi: 10.1371/journal.pcbi.1007127 (PMC6583959; doi:10.1371/journal.pcbi.1007127)
Supplement: S1 Table — If sufficient nutrient is available, cells consume nutrients at the maximum rate. At low nutrient concentrations, the amount is divided equally between all cells residing within a node, resulting in uptake rates lower than the maximum uptake rate. (DOCX) [file pcbi.1007127.s005.docx]

| COMETS comparison | Lactose | Oxygen | Acetate | Methionine |
| --- | --- | --- | --- | --- |
| *E. coli* K-12 (iJO1366) | 10 | 30 | 10 | 1 |
| *S. enterica* LT2 (iRR1083) | 0 | 30 | 10 | 1 |
| Micromodels | **Citrate** | **Oxygen** | **Acetate** | **Nitrate** |
| *P. putida* KT2440 (iJN746) | 10 | 30 | 10 | 0 |
| *P. stutzeri* A1501 (iPB890) | 10 | 30 | 10 | 30 |
| *Glucose perfusion* | **Glucose** | **Oxygen** |  |  |
| *P. stutzeri* A1501 (iPB890) | 10 | 30 |  |  |
| *Strategic variation* | **Glucose** | **Oxygen** | **Acetate** | **Nitrate** |
| *P. stutzeri* A1501 (iPB890) | 10 | 30 | 10 | 30 |

S1 Table: Summary of maximum uptake rates imposed for all metabolic networks and simulation scenarios. If sufficient nutrient is available, cells consume nutrients at the maximum rate. At low nutrient concentrations, the amount is divided equally between all cells residing within a node, resulting in uptake rates lower than the maximum uptake rate.
