# Supplementary material for: Modeling metabolic networks of individual bacterial agents in heterogeneous and dynamic soil habitats (IndiMeSH)
Source: PLoS Comput Biol. 2019 Jun 19;15(6):e1007127. doi: 10.1371/journal.pcbi.1007127 (PMC6583959; doi:10.1371/journal.pcbi.1007127)
Supplement: S2 Table — This table contains a list of all parameters that can be set by the user for simulations containing multiple substrates and species. The list is exhaustive excluding the actual topology of the pore network (i.e. node coordinates, adjacency matrix and boundary location). (DOCX) [file pcbi.1007127.s006.docx]

| Parameter | Description |
| --- | --- |
| Δt | Time step [s] |
| T | Total simulated time [s] |
| Ψ | Matric potential for the whole simulation or each time step [Pa] |
| dim_1,i_ | First dimension for each individual pore i (radius of inscribed circle for triangles [m], width for rectangles [m]) |
| dim_2,i_ | Second dimension for each individual pore i (central angle for triangles [°], height for rectangles [m]) |
| C_0,n,s_ | Initial concentration for each substrate s at each node n [mol/m^3^] |
| C_bnd,s_ | Boundary concentration at each boundary bnd and substrate s [mol/m^3^] |
| D_s_ | Diffusion coefficient for each substrate s [m^2^/s] |
| w_b_ | Weight of an individual cell for each species b [kg] |
| p_0,b_ | Tumbling probability in absence of chemotaxis for each species b [-] |
| v_0,b_ | Swimming velocity in pure water for each species b [m/s] |
| Χ_b_ | Chemotactic sensitivity for each species b [m^2^/s] |
| υ_b,s_ | Maximum uptake rate of substrate s for species b [mmol/gDW/h] |

S2 Table: List of parameters that can be set by the model user. This table contains a list of all parameters that can be set by the user for simulations containing multiple substrates and species. The list is exhaustive excluding the actual topology of the pore network (i.e. node coordinates, adjacency matrix and boundary location).
