# Supplementary material for: Modeling metabolic networks of individual bacterial agents in heterogeneous and dynamic soil habitats (IndiMeSH)
Source: PLoS Comput Biol. 2019 Jun 19;15(6):e1007127. doi: 10.1371/journal.pcbi.1007127 (PMC6583959; doi:10.1371/journal.pcbi.1007127)

# IndiMeSH: Input file description

## Overall specifications: SimulationSpec.txt

The main parameters of the model are set in the SimulationSpec.txt file. This file contains a header row and four numbers. The first number contains the dimension of the physical domain unit (exclusively being 1 representing linked one dimensional pores, plans to expand to two dimensional patches). The second value represents the dimension of each pore and can be set to 4 (when pores are represented with rectangular cross-sections) or 3 (when represented by triangular cross-sections). The third number contains the number of chemical substrates present in the simulation while the fourth number represents the total number of species in the simulation.


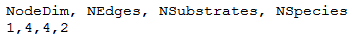


## Temporal domain

### Description of Temporal.txt

This file contains all the information concerning temporal aspects of the model. The file includes a header line and three values. The time step for each iteration, the total simulated time, and the interval at which the simulation will be saved as a mat file. All units are in seconds.


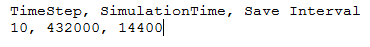


## Physical and hydrological domain

### Description of NodeCoordinates.txt

This file contains the spatial coordinates of the nodes representing the pore network. The size of the file needs to be [number of points] x 3. The three columns represent the X,Y and Z coordinates in meters.


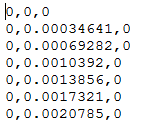


### Description of NodeAdjacency.txt

This file contains all information about the connectivity between nodes. The dimension of the file needs to be [number of pores] x 3. The three columns can easily be created in Matlab using the find() function on a sparse or dense adjacency matrix.


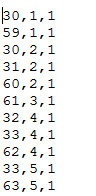


### Description of NodeBoundaries.txt

This file contains all information concerning the location of boundary conditions and the typo of boundary. The number of boundaries are defined by the number of columns. The number of rows depends on the maximum number of nodes included in a boundary (+1 for the header row). All rows that are not required in the matrix (because the respective boundary in the column has fewer nodes included) are set to 0. The first row contains the information if a boundary is considered to be an air boundary (1) or not (2). Any boundary that is considered to be an air boundary (i.e. the nutrients supplied at this boundary are via the gas phase) propagates the source into the network in unsaturated conditions. I two nutrients, one aqueous and one gaseous, are located at the same positions, two boundaries need to be defined with the same node ID but one containing 0 in the header (for aqueous boundary) and the other with 1 (for the gaseous boundary).


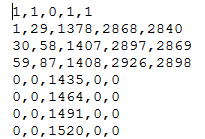


### Description of Geometry.txt

This file contains the information about pore geometry. The dimension of the matrix is [number of pores] x 2. For rectangular pores, the first column defines the pore height whilst the second contains the pore width. For triangular pores, the first column defines the inscribed circle radius whilst the second pore contains the central angle degree. All units are in meters and degrees. The file contains a header row which defines the columns.


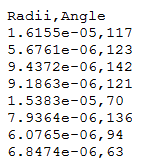


### Description of HydrationBoundary.txt

This file contains the information which nodes are in direct contact with air for the calculation of air penetration into the network. When updating the hydrological domain, these nodes are considered to be in direct contact with air and are used as starting points for a burning type algorithm to considered how far air penetrates into the pore network. The dimension of the matrix is [number of nodes in contact with air] x 1.


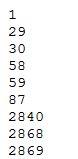


### Description of Hydration#.txt

This file determines the matric potential of the simulation. It can consist of either a single number (which is then used for the whole simulation, steady state concerning hydrology) or an individual value for each time step. In the latter case, the dimension of the vector needs to be [number of iterations] x 1. The unit of the matrix potential is Pa. In the example below, the matric potential is set to -1 Pa for the whole simulation (i.e. fully saturated). There can be multiple hydration text files named Hydration1.txt, Hydration2.txt etc. The number of hydration files corresponds to the number of hydration conditions set in the initialisation file. These simulations can be run in parallel if the parallel option is set to “simulations”.


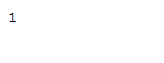


## Chemical domain

### Description of SubstrateSpec.txt

This file contains the general information of the substrates used. The dimension of the matrix is [number of substrates] x 2 excluding the header row. The first column includes the names of the substrate (this is primarily to facilitate visualisation of the results). The second column contains the diffusion coefficient of the substrate in water using the units of m^2^/s. If the diffusion coefficient is set to 0, the concentration set in InitialConc.txt is set homogeneously across the domain (in this case the dimension of the file needs to be 1 x [number of substrates] as explained below).


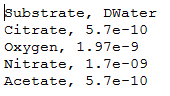


### Description of InitialConc.txt

This file contains information on the distribution of substrates at the beginning of a simulation. Excluding the header row, the dimension of the matrix is either 1 x [number of substrates] or [number of nodes] x [number of substrates]. In the first case, the value is set for the whole domain (e.g. to set the concentration to zero across the whole domain). In the second case a value is set for each not which allows to set predetermined gradients across the domain. The units of each value is in mM (which equals mol/m^3^ to remain consistent with SI units).


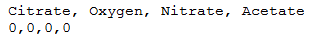


### Description of Boundary#.txt

This file contains the information concerning boundary conditions for each substrate. The number of files required reflects the number of columns defined in the NodeBoundaries.txt file. The dimension of the file is either 1 x [number of substrates] or [number of iterations] x [number of substrates]. In the first case, the conditions are set at the respective boundary for the whole simulation whilst the latter case enables to set different conditions for each time step (e.g. a declining source during the simulation period). A negative value (here -1) indicated a no flux boundary. A positive value creates a constant concentration boundary condition. A sink can be introduced by setting the value to 0. The units of each value is in mM (which equals mol/m^3^ to remain consistent with SI units).


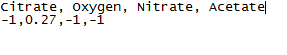


## Biological domain

### Description of SpeciesSpec.txt

This file contains the general description of the species present. Besides the header row, each row contains information for a single species. The columns contains (in order) the species genus, name, shape, metabolism type and motility type. Currently, the shape of the cells does not influence the simulation (would be included in cell shape dependent shoving algorithms) and only swimming motility is implemented. The metabolism type can be either FBA or Monod (where the implementation of the latter is not described in detail here).


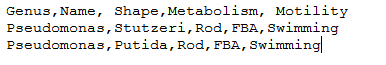


### Description of GeneralSpecies#.txt

This file contains general information about the species. An individual file is required for each species present in the simulation. Four numbers are required specified by the header row: the weight of an individual cell in kg, the metabolic network name (this has to coincide exactly with the name given to the mat file in the MetabolicNetworks folder), the FBA objective function ID and biomass function ID. In this case, the ID is the location of the respective reaction in the metabolic networks rxns vector.


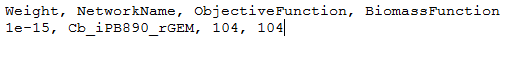


### Description of MotilitySpecies#.txt

This file contains general information about the species motility. An individual file is required for each species present in the simulation. The file contains three parameters indicated by the header row: the tumbling probability per time step, the swimming velocity in one dimension in m/s and the chemotactic sensitivity in m^2^/s.


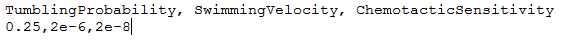


### Description of MetabolismSpecies#.txt

This file contains general information about the species metabolism. An individual file is required for each species present in the simulation. The file contains two rows, the first with the maximum uptake rate for each metabolism and the second with the ID of the uptake reaction in the metabolic network. In this case, the ID is the location of the respective reaction in the metabolic networks rxns vector. The dimension of the file excluding the header row is thus [number of substrates] x 2. If a species cannot use a specific substrate, set both the uptake rate and ID to 0.


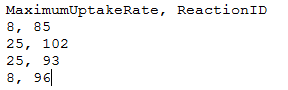


### Description of InoculationSpec#.txt

This file contains the location and number of inoculated cells for all species. The dimension of the matrix is [number of nodes inoculated] x [number of species + 1]. The first row contains the node ID which is inoculated with the number of cells indicated by the following columns of the same row. Multiple inoculation files can exist named InoculationSpec1.txt, InoculationSpec2.txt etc. The number of inoculation files corresponds to the number of inoculations set in the initialisation file. These simulations can be run in parallel if the parallel option is set to “simulations”.


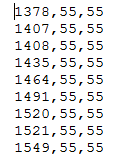

Supplement: S1 Data — (ZIP) [file pcbi.1007127.s007.zip › IndiMeSH_Suite/IndiMeSH_InputFileDescription.docx]
